# Efficient generation of *B2m*-null pigs via injection of zygote with TALENs

**DOI:** 10.1038/srep38854

**Published:** 2016-12-16

**Authors:** Yong Wang, Yinan Du, Xiaoyang Zhou, Lulu Wang, Jian Li, Fengchao Wang, Zhengen Huang, Xingxu Huang, Hong Wei

**Affiliations:** 1 Department of Laboratory Animal Science, College of Basic Medical Sciences, Third Military Medical University, Chongqing 400038, China; 2 MOE Key Laboratory of Model Animal for Disease Study, Model Animal Research Center of Nanjing University, National Resource Center for Mutant Mice, Nanjing 210061, China; 3 School of Life Science and Technology, ShanghaiTech University, 100 Haike Rd., Pudong New Area, Shanghai 201210, China; 4 Department of Immunology, College of Basic Medical Sciences, Third Military Medical University, Chongqing 400038, China; 5 Institute of Combined Injury, College of Military Preventive Medicine, Third Military Medical University, Chongqing 400038, China; 6 Research Institute of Burns, Southwest Hospital, Third Military Medical University, Chongqing 400038, China

## Abstract

Donor major histocompatibility complex class I (MHC I) molecules are the main targets of the host immune response after organ allotransplantation. Whether and how MHC I-deficiency of pig donor tissues affects rejection after xenotransplantation has not been assessed. Beta2-microglobulin (B2M) is indispensable for the assembly of MHC I receptors and therefore provides an effective target to disrupt cell surface MHC I expression. Here, we report the one-step generation of mutant pigs with targeted disruptions in *B2m* by injection of porcine zygotes with *B2m* exon 2-specific TALENs. After germline transmission of mutant *B2m* alleles, we obtained F1 pigs with biallelic *B2m* frameshift mutations. F1 pigs lacked detectable B2M expression in tissues derived from the three germ layers, and their lymphocytes were devoid of MHC I surface receptors. Skin grafts from B2M deficient pigs exhibited remarkably prolonged survival on xenogeneic wounds compared to tissues of non-mutant littermates. Mutant founder pigs with bi-allelic disruption in *B2m* and B2M deficient F1 offspring did not display visible abnormalities, suggesting that pigs are tolerant to B2M deficiency. In summary, we show the efficient generation of pigs with germline mutations in *B2m*, and demonstrate a beneficial effect of donor MHC I-deficiency on xenotransplantation.

Pigs are considered promising candidate donors for xenotransplantation because they share many anatomical and physiological features with humans, and have a large litter size and a relatively short gestational period. Their greater phylogenetic distance to human versus other potential donor organisms such as non-human primates may be associated with lower risk for cross-species disease transmission^1^. Rejection of the graft by immune-mediated mechanisms represents one of the main barriers to successful xenotransplantation. Various strategies have been pursued to genetically modify pigs to reduce immune incompatibility and to prevent host versus donor immune rejection after xenotransplantation. Approaches include pigs with transgenic expression of human proteins regulating the activation of complement, T cells, NK cells or other components of the immune system^2^,^3^,^4^,^5^,^6^,^7^,^8^, and pigs lacking a functional glycoprotein alpha-galactosyltransferase 1 (GGTA1) gene, which encodes the enzyme responsible for the expression of the immunogenic alpha 1,3-Gal epitope in non-primate species^9^,^10^.

Major histocompatibility complex class I (MHC I) peptides are cell surface molecules encoded by a large family of highly polymorphic genes. MHC receptors play a major role in the immune system of all vertebrates, and are the main target of the early alloimmune response after organ transplantation. Pig MHC I molecules have been classified into two groups^11^,^12^,^13^. The first group, termed swine leukocyte antigen 1a (SLA-1a), encompasses highly polymorphic and widely expressed proteins that are commonly recognized as the functional orthologues of the human classical MHC I antigens (HLA-A, HLA-B, and HLA-C). Proteins of the second group, SLA-1b, have been classified as non-classical MHC I antigens because these molecules exhibit limited polymorphism and restricted expression patterns, and their orthology or functional homology to human non-classical MHC I antigens, such as HLA-E, HLA-F and HLA-G, remains to be established^13^.

Human CD8+ positive cytolytic cell subpopulations as well as NK cell subpopulations have been shown to directly recognize SLA-1a molecules, leading to the lysis of target cells^14^,^15^. However, the relevance of porcine MHC I molecules in xenotransplantion is not fully understood and needs to be further explored by transplantation experiments with MHC I-deficient donor pig tissues. Porcine MHC I is a heterotrimeric complex consisting of a heavy α-chain, a light β-chain termed β2-microglobulin (B2M), and short peptides. The α-chain is highly polymorphic and is encoded by several genes that exist inmultiple allelic variants. In contrast, the B2M molecule, which is indispensable to MHC I assembly on the cell surface, is non-polymorphic and encoded by a single gene. Therefore, *B2m* provides a simple and effective target to disrupt porcine MHC I expression on the cell surface.

Transcription activator-like effector nucleases (TALENs) are versatile genomic editing tools that have been successfully used in different species, including pigs^16^,^17^,^18^,^19^,^20^,^21^,^22^,^23^. More recently, clustered regularly interspersed short palindromic repeats (CRISPR)/CRISPR-associated 9 systems (CRISPR/Cas9) have been developed that can mediate effective genome editing in a range of species^24^,^25^,^26^,^27^,^28^. CRISPR/Cas9 systems can be readily modified and synthesized and allow for multiplex editing, whereas TALENs offer higher sequence specificity and are associated with a lower frequency of off-target effects^29^. Here, we used TALEN technology to obtain a targeted disruption of pig *B2m.* Pigs harboring frameshift mutations in *B2m* were efficiently generated via cytoplasmic injection of zygotes with TALEN mRNA. Following germline transmission of mutant alleles, we obtained *B2m* null-mutant pigs, which were devoid of B2M in tissues derived from all three germ layers and lacked MHC I assembly on the cell surface of lymphocytes. These MHC I-deficient pigs can be used as donors to investigate the biological effects on xenotransplantation, and also provide a non-rodent model to better explore how MHC I receptors regulate immunity in various species.

## Results and Discussion

### Design and validation of TALENs targeting *B2m*

**Figure 1:**
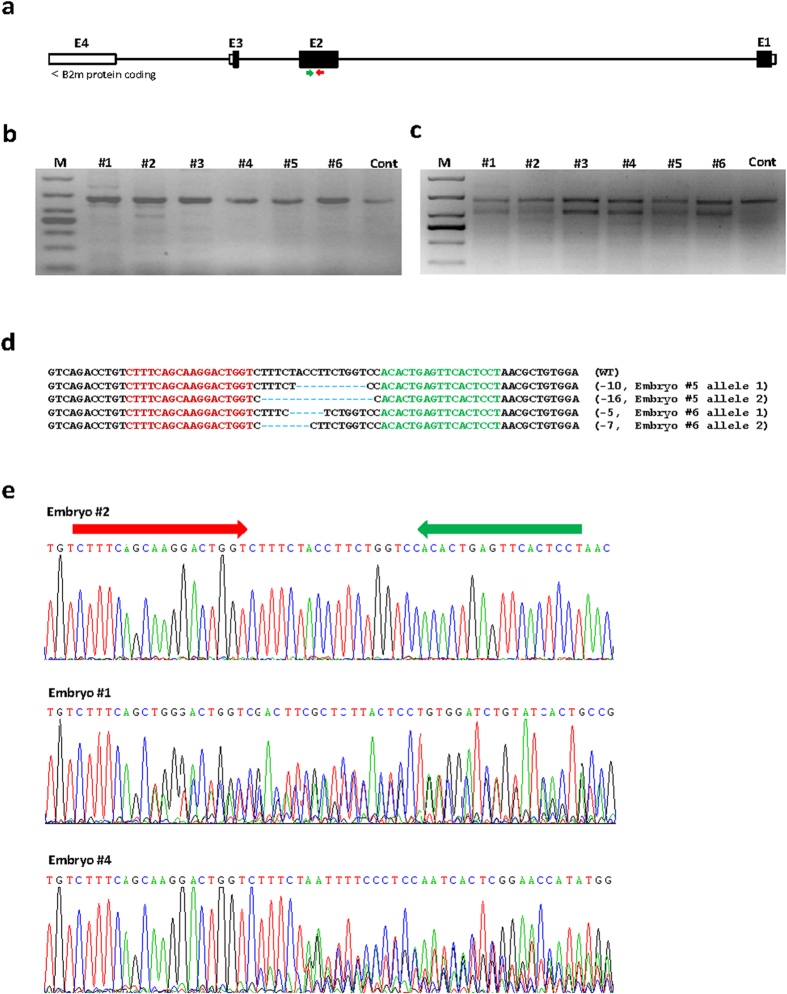
Evaluation of TALEN-mediated modification of *B2m* in pig parthenogenetic embryos. (**a**) Schematic diagram of pig B2M protein coding region and the targeting locus of *B2m*-TALENs. Red arrow and green arrow indicate left arm and right arm of *B2m*-TALENs, respectively. (**b**) The PCR products covering *B2m*-TALEN target site amplified from genomic DNAs of individual injected PA embryos. #1~#6 are the parthenogenetically activated(PA) embryos injected with *B2m*-TALEN mRNAs, and Cont is the wild-type PA embryo as control. (**c**) T7EN1 cleavage assay of PCR products covering the targeting site (**b**). #1~#6 are the parthenogenetically activated(PA) embryos injected with *B2m*-TALEN mRNAs, and Cont is the wild-type PA embryo as control. (**d**) Sequencing results of the modified *B2m* alleles detected in pig parthenogenetic embryos. Embryo #5 and #6 are two examples. Sequences complementary to left arm and right arm of *B2m*-TALENs are labeled in red and green; mutations, blue, lower case. (**e**) Chromatographs of sequencing modified *B2m* alleles in parthenogenetic embryos in which overlapped peaks were observed. Embryo #2 is a wild-type sample as control and #1 and #4 are two examples with overlapped peaks near targeting site. Red arrow and green arrow indicate the targeting sites of *B2m*-TALEN left arm and right arm, respectively.

To disrupt B2M expression in pig, we designed a pair of TALEN molecules that target exon2 of pig *B2m* (Fig. 1a). These *B2m*-TALENs did not have any predicted off-target sites (OTs) in the pig genome according to UCSC In-Silico PCR software (http://genome.ucsc.edu/cgi-bin/hgPcr). To assess *B2m*-TALEN-affected early stage development in pig embryos, we injected mRNAs encoding the TALEN pair (10 ng/mL each) into the cytoplasm of parthenogenetically activated (PA) pig embryos at the one-cell stage, followed by culture of embryos *in vitro* to the blastocyst stage. Injected PA embryos exhibited similar rates of cleavage and development to the blastocyst stage as control (untreated) PA embryos (Table 1). The slightly higher rate of blastocyst formation in microinjected embryos may be related to an increased influx of Ca^2+^ during micromanipulation, and is consistent with our previous observations of preimplantation development in Cas9/sgRNA-injected pig PA embryos^28^.

To assess for TALEN activity at the *B2m* target locus, we isolated genomic DNA from individual embryos and employed the T7 endonuclease I (T7EN1) cleavage assay that detects heteroduplex DNA in PCR products amplified across the target site. T7EN1 cleavage bands were identified in 5 of 6 PA embryos (Fig. 1b,c). Sequencing of PCR products from the 5 embryos with T7EN1 cleavage bands identified indels and overlapping peaks in sequencing chromatographs (Fig. 1d,e; Table 2), indicating that the *B2m*-TALENs cleaved the target site. These data demonstrate that TALENs targeting pig *B2m* had no adverse effects on pig embryo development *in vitro*, and exhibited target-specific nuclease activity.

### One-step generation of *B2m* knockout pigs via injection of zygotes with TALEN mRNAs

**Figure 2:**
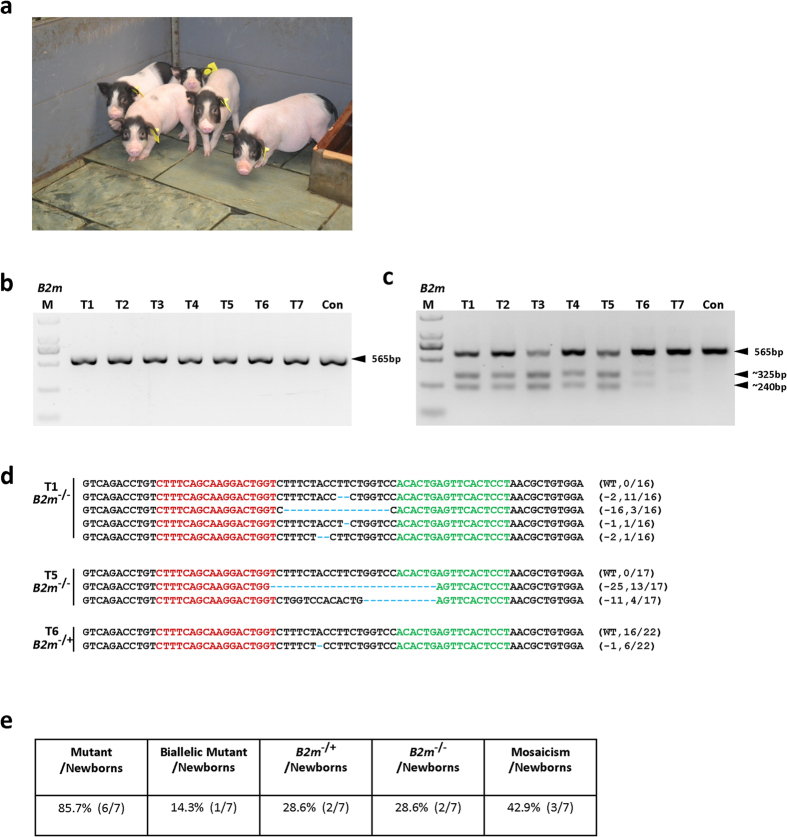
Detection of *B2m*-TALEN-mediated modifications of *B2m* in founder pigs. (**a**) A representative photo showing 52-day-old founder pigs carrying *B2m* mutations. (**b**) PCR products of the targeted region of *B2m* from founder pigs co-microinjected with a pair of TALEN mRNA. Eight piglets were born and one died soon after birth. The seven alive founders were named from T1 to T7. Con denotes wild-type pig as control. (**c**) Detection of TALEN-mediated on-target cleavage of *B2m* by T7EN1 cleavage assays. All PCR products from (**b**) were subjected to T7EN1 cleavage assays. All the founders except T7 could be digested by T7EN1 which suggests that these founders carry *B2m* mutations. (**d**) Sequencing results of modified *B2m* alleles detected in founder pigs. At least 16 TA clones of the PCR products were analyzed. Sequences targeted by left and right TALEN are labeled in red and green respectively. The mutations in blue, lower case; deletions (−), N/N indicates positive colonies out of total sequenced. See also Figure S1. (**e**) Summary of generated *B2m* mutant pigs by TALENs. Founders showed more than two genotypes are considered as mosaicism.

We chose to pursue the *B2m* knockout pig model in the Chinese Bama minipig breed. The Bama minipig is widely used as a large animal model for biomedical research, has a relatively high degree of genetic stability, and is relatively higher inbred. A total of 118 fertilized 1-cell stage embryos were recovered from the oviducts of mated sows, followed by cytoplasmic microinjection of *B2m* TALEN mRNAs (10 ng/mL each) and transfer of injected zygotes into eight estrus-synchronized recipient sows. Three recipients became pregnant, and one aborted. The remaining 2 recipient sows delivered 7 full-term piglets (Fig. 2a, Table 3). We isolated genomic DNA from ear punch tissue from the 7 piglets and amplified the genomic region surrounding the *B2m* TALEN target site by PCR. In all samples, a single band was obtained (Fig. 2b), and T7EN1 cleavage bands were detected in PCR samples of 6 of the 7 animals (Fig. 2c). Sequencing of the PCR products revealed indel mutations in the individuals with T7EN1 cleavage bands as expected (Fig. 2d, Fig. S1). Of the 6 mutant founder pigs, bi-allelic frameshift mutations were detected in 1 founder (#T5), mono-allelic null mutations in 2 founders (#T4 and #T6), and mosaicism with at least 3 different genotypes in 3 founders (#T1, #T2 and #T3) (Fig. 2e, Table 4). Interestingly, no in-frame mutation was detected in any of the founders (Fig. 2e, Fig. S1 and Table 4), suggesting a potential bias towards double strand break (DSB)-induced mutations at this site.

### Germline transmission of mutant *B2m* alleles

**Figure 3:**
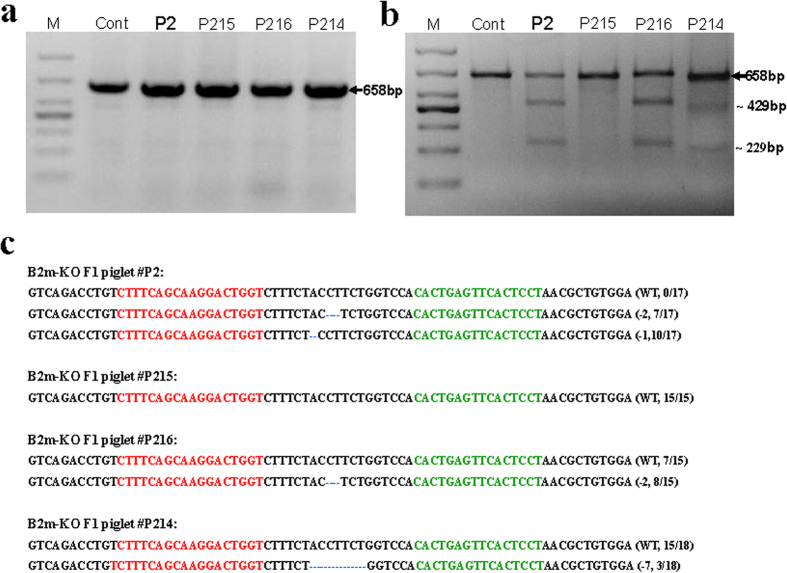
Genetic analysis of the four offspring piglets of F1 generation derived from the founder T#1(♀) and #T6(♂). (**a**) PCR amplification of the region around target site in *B2m* using the primers: B2m-TAL-F: 5-CGGTGAAATCCTCTGGCG-3; B2m-TAL-R: 5-GCCTGTGCTTCCCTGAGACT-3; product size: 658bp; M: DNA marker DL1000; WT: wild-type pig genomic DNA; P2, P215, P216 and P214 indicate the piglet #P2, piglet #P215, piglet #P216 and piglet #P214, respectively; Cont: wild type pig as control. (**b**) All the PCR products from F1 piglets subjected to T7EN1 cleavage assay. (**c**) Sequencing results of the modified *B2m* alleles in the four piglets of F1 offspring; the sequences targeted by left and right TALEN are labeled in red and green respectively. The mutations in blue, lower case; deletions (−). N/N indicates the positive colonies out of total sequenced.

The two founder pigs (#T1 and #T5, Table 4), in which frameshift, but no other types of mutations were detected and affected both alleles, indicated by the absence of a wild-type allele, did not exhibit any obvious phenotypic abnormalities and grew normally into adults, suggesting that the lack of a B2M molecule had no apparent adverse effects on the development or health of these animals. However, gene-modified founder animals produced by the injection of customized engineered endonucleases into zygotes often exhibit mosaicism for the targeted mutations^20^,^23^,^25^,^28^. Therefore, genetically unmodified cells may be present in various tissues of the founders and functionally compensate for the *B2m* null mutant cells, masking potential phenotypic manifestations. To address whether pigs were truly tolerant of B2M-deficiency and whether the *B2m* mutations could be transmitted through the germline, we mated the female founder pig #T1, harboring three different frameshift mutations and no wild type target sequence according to ear tissue analysis, with the male founder #T6, which should produce offspring with bi-allelic *B2m*-null mutations. We obtained 4 piglets in the F1 generation (F1 piglets) and performed genetic analysis as before. No additional bands were detectable in PCR reactions amplifying across the *B2m*t arget area, and T7EN1 cleavage bands were detected in 3 of 4 F1 offspring (Fig. 3a,b). Sequencing of the PCR products revealed that of the 4 F1 piglets, 1 (#P2) was bi-allelically mutant with two frameshift mutations, 2 (#P214 and #P216) were mono-allelically mutant with one frameshift mutation and the remaining piglet (#P215) did not carry a *B2m* mutation (Fig. 3c). The F1 piglet with bi-allelic frameshift *B2m* mutations (#P2) had a normal appearance and developed into a healthy adult. Of the 5 mutations that were detected in total between the 2 founder animals (#T1 and #T6), only 1 (−1 bp (A)) was present in the F1 offspring. Lack of germline transmission of the other mutations may either be due to chimerism in the germline, or that the litter size of 4 piglets was not large enough for detection of those mutations to be transmitted through the germline. Furthermore, two novel *B2m* mutations (−7 bp and −2 bp (CT)) were detected in the F1 piglet #P214, #P216 and #P2, whereas piglet #P215 lacked targeted *B2m* mutations. The presence of novel mutation(s) or absence of known mutation(s) of founders in individual offspringis were likely due to the high genetic mosaicism in the founder animals. As a result, different genotypes may exist in different tissues, and therefore a fraction of gametes in the founders may contain novel mutant allele(s) or the wild type allele, which were not detected in the ear tissues used for genetic screening of founders. Taken together, these data demonstrate germline transmission of the null mutations introduced into pig *B2m* via injection of zygotes with TALENs and suggest that pigs tolerate the lack of B2M.

### Phenotypic analysis of *B2m*-null pigs

**Figure 4:**
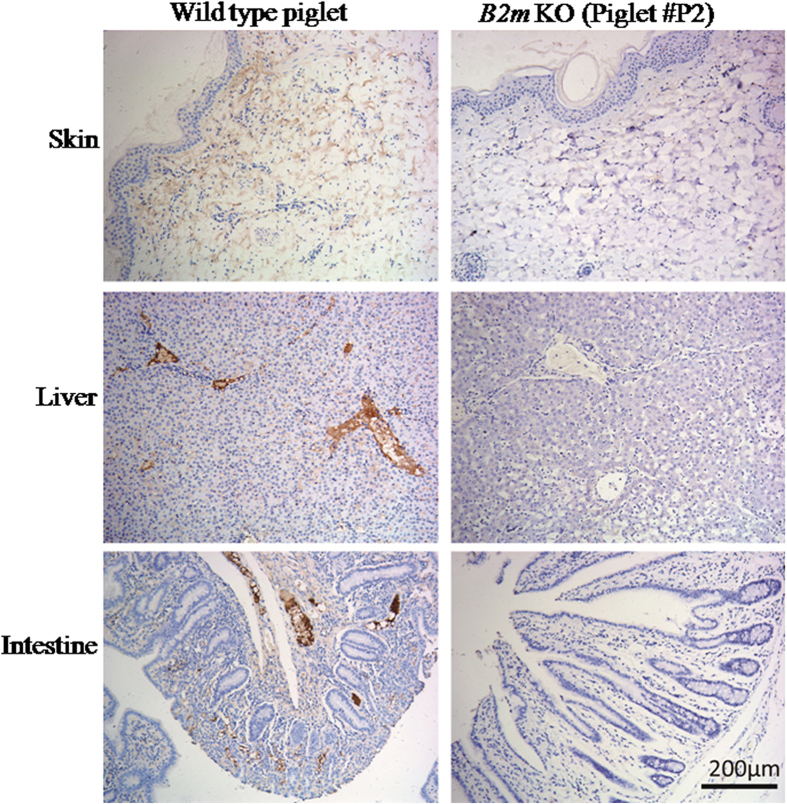
Immunohistochemical analysis of *B2m* expression in the F1 piglet #P2 with bi-allelic mutation. The antibody specific porcine B2M molecule (LifeSpan, LS-B1858) was used to detect *B2m* expression in skin, liver and intestine which are derived from three different germ layers. WT: wild type pig samples as control.

**Figure 5:**
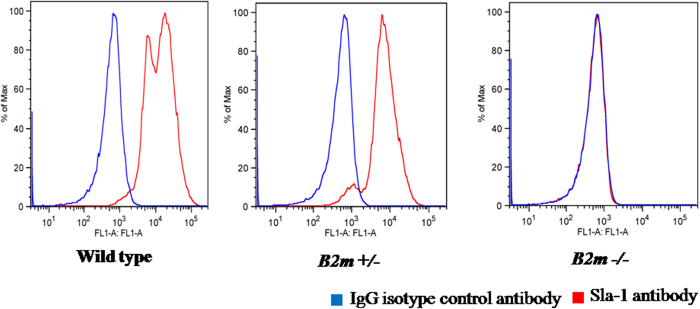
FACS assay of peripheral lymphocytes from the F1 piglets stained with FITC-conjugated SLA1-antibody. Fresh peripheral lymphocytes were collected from the piglets with bi-allelic mutation, mono-allelic mutation and no mutation (wild type pig), and stained with FITC-conjugated SLA-1 antibody and FITC-conjugated IgG isotype control antibody in parallel. The red histogram is for lymphocytes stained with SLA-1 antibody, and the blue one for IgG isotype control antibody.

Germline transmission of mutant *B2m* alleles provided an opportunity to verify absence of the B2M protein in various tissues and to evaluate the functional consequences of *B2m* null mutations on MHC I assembly and immune rejection after xenotransplantation. Using a monoclonal antibody specific for pig B2M protein, we performed immunohistochemical staining to investigate B2M expression in skin, liver, and intestine, which are derived from different germ layers. As shown in Fig. 4, B2M was not detectable in tissues from the F1 pig (#P2) with bi-allelic *B2m* mutations, but detected in wild-type control tissues, indicating that the frameshift *B2m* mutations effectively disrupted B2M protein expression. To further investigate whether the lack of B2M expression eliminated or reduced pig MHC I assembly on the cell surface, we collected peripheral lymphocytes from F1 animals and performed fluorescence-activated cell sorting (FACS) analysis of samples stained either using a FITC-conjugated mouse monoclonal antibody specific for pig MHC I (SLA-1) or, as a negative control, with a FITC-conjugated mouse IgG isotype antibody without known binding specificity. As shown in Fig. 5, the SLA-1 histogram of the sample from the F1 pig with bi-allelic null mutations of *B2m* completely overlapped with the isotype control peak, whereas distinct peaks were present in samples from a wild-type pig and a mutant pig with mono-allelic *B2m* deletions. These data indicate that lymphocytes from the mutant pig with biallelic *B2m* frameshift mutations were devoid of MHC I complexes on the cell surface. Interestingly, the SLA-1 peak of the sample from the pig with mono-allelic *B2m* deletions was shifted in intensity towards the isotype control peak, suggesting that haploid expression of B2M also reduced MHC I complex assembly. Thus, availability of B2M may affect pig MHC I assembly on the cell surface in a dose-dependent manner, similar to findings in *B2m* knockout mouse lymphocytes^30^. The above data demonstrated that the frameshift mutations caused by *B2m*-TALENs effectively disrupted B2M expression, and eliminated pig MHC I assembly on cell surface.

**Figure 6:**
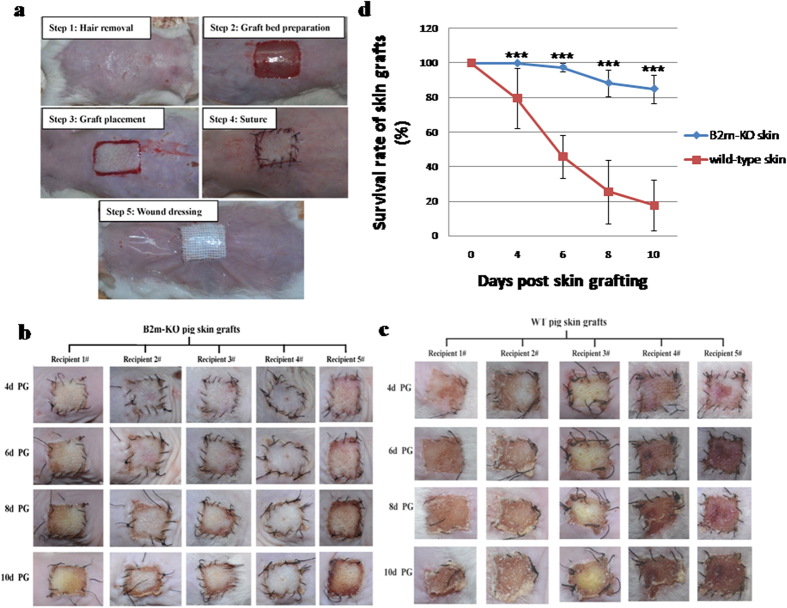
Survival of skin grafts. Skin grafts were prepared from the bi-allelically mutant F1 pig (#P2) and the F1 pig (#P214) without mutation of the same litter, respectively, and grafted into wounds made on the back of mice of FVB/N inbred strain. (**a**) The procedure of skin grafting. (**b**) The skin grafts of the F1 *B2m*-null pig (#P2) on different days post grafting; PG: post grafting. (**c**) The skin grafts of the F1 pig containing wild type target sequence (#P214) on different days post grafting; PG: post grafting. (**d**) Skin graft survival rates on different days post grafting; *indicates statistical significance.

To assess the functional consequences of the absence of MHC I complex in pig tissues on xenotransplantation, we performed skin grafting from pigs to mice as described previously^31^,^32^. For improved graft survival, grafted skin pieces were covered with a dressing of several layers of gauze pieces and secured with a transparent film dressing (Fig. 6a). Skin grafts from the F1 bi-allelically mutant pig (#P2) exhibited remarkably prolonged survival compared to those from the littermate F1 pig lacking *B2m* mutation (#P214) (Fig. 6b,c and d). On day 6 post grafting, the majority of grafts without *B2m* mutation exhibited necrosis, and grafts were almost completely rejected on day 10 post grafting, demonstrating that acute immune rejection against grafts occurred (Fig. 6c). In contrast, the majority of skin grafts with bi-allelic *B2m* mutations exhibited no detectable necrosis on day 10 post grafting (Fig. 6b). These data suggest that donor MHC I complex deficiency may be beneficial for xenotransplantation. However, compatibility may require the additional elimination of the genes encoding the alpha heavy chain of pig MHC I, i.e. pig classical MHC I molecules (SLA-1, 2, 3), to completely prevent intracellular MHC I expression. While this manuscript was in preparation, pigs with disruption of the classical MHC I molecules (SLA-1, 2 and 3) were reported^33^. However, the presence of a porcine *B2m* gene in these pigs would hamper any attempted humanization of MHC I, because endogenous porcine B2M could combine with human MHC I heavy alpha chains in hybrid complexes as observed in rodent models^34^. Furthermore, the presence of pig non-classical MHC I molecules would be immunogenic after xenotransplantation. Therefore, the combined disruption of B2M and MHC I alpha heavy chains in pigs will be required to investigate the functional consequences of pig MHC I deficiency on xenotransplantation. Such MHC I-null pigs could be used for MHC I humanization with transgene encoding the human counterparts, especially non-classical MHC I molecules such as HLA-G, HLA-E and HLA-F, which are of limited immunogenicity for the human immune system due to their limited polymorphism and inhibitory function on human NK cell activation.

In summary, we demonstrate the efficient generation of *B2m*-null pigs after germline transmission of mutant alleles that were generated by the injection of zygotes with TALENs targeting exon2 of *B2m*. We find that the absence of B2M protein prohibits the assembly of MHC I on the cell surface but has no detectable adverse effects on pig development and health. Furthermore, our study also showed a possible beneficial effect of donor MHC I complex deficiency on xenotransplantation success.

## Materials and Methods

### Animals

The animals used in this study were regularly maintained in the Laboratory Animal Center of the Third Military Medical University. All the protocols involving the use of animals were in accordance with approved guidelines of the Institutional Animal Care and Use Committee of the Third Military Medical University (Approval ID: SYXK-PLA-2007036).

### Vector constructs and *in vitro* transcription

*B2m*-TALENs were designed using TALEN-NT software and constructed by Golden Gate methods as previously described^35^. pCS2-PEAS and pCS2-PERR were utilized as upstream and downstream TALEN-assembling backbones, respectively, as described^36^. The CDS region of the TALEN vectors were cut out using SpeI and NheI, and subcloned into pcDNA3.1 vector to be under the drive of T7 promoter. The TALEN-expressing plasmid was linearized using PmeI, of which the cutting site was located at the 3′-end of the FokI domain. *B2m*-TALEN mRNA was prepared via *in vitro* transcription using the linearized plasmid as the templates with T7 U1tra Kit (Ambion, Austin, TX), and further purified by RNeasy Mini Kit (Qiagen).

### TALEN efficacy test via pig parthenogenetic embryo injection

The efficacy of *B2m* TALENs was tested in pig parthenogenetically activated (PA) embryos. To prepare pig PA embryos, the cumulus-oocyte complexes (COCs) were collected from slaughter house and cultured for *in vitro* maturation as described^10^. The mRNAs of the designed TALEN pair were mixed and diluted to be the final concentration of 10 ng/uL each using RNase-free deionized water. The pig oocytes were freed of cumulus, and the matured oocytes with extruded polar body were selected out and subjected to cytoplasmic microinjection with diluted TALEN mRNAs as described^28^,^37^. The injected oocytes were activated by direct current electrical pulses (1.2 KV/cm, 30 μs, two times, 1 sec interval) and the activated oocytes (PA embryos) were cultured in PZM-3 media as described by Wang *et al*.^37^. The cleavage rate of PA embryos was counted at 48 h post activation and blastocystes were harvested at 144 h post activation. Pig genomic DNA was extracted from individualPA blastocysts by incubating individual embryos in lysis buffer as described^28^,^37^. Using the genomic DNAs as templates, a primer pair set (B2m-TAL-F: 5-CGGTGAAATCCTCTGGCG-3; B2m-TAL-R2: 5-GCCTGTGCTTCCCTGAGACT-3; product size: 658bp) were used to amplify modified *B2m* alleles in injected embryos by PCR, and the amplification products were subjected to T7 endonuclease 1 (T7EN1, NEB) cleavage assay or Sanger sequencing after purification using gel extraction kit (Qiagen).

### Production of *B2m* knock-out pigs via zygote injection with TALENs

The TALEN mRNAs were mixed and diluted as described above. Pig zygotes were surgically collected from the oviducts of mated sows. The collected zygotes were subjected to cytoplasmic microinjection with the diluted TALEN mRNA mixture in the same way as that for parthenogenetic embryos as described above. Shortly after injection, the injected zygotes were transferred into estrus-synchronized foster mother sows as described^28^,^37^. Pregnancy was investigated by observing the oestrus behaviors of recipient sows at every ovation circle.

### T7EN1 cleavage assay and sequencing

Pig tissue samples were digested in lysis buffer (0.4 M NaCl, 2 mM EDTA, 1% SDS, 10 mMTris-HCl, and100 mg/ml Proteinase K) overnight. Genomic DNAs were extracted from the lystes after treatment with phenol-chloroform and recovered via alcohol precipitation. For performing T7EN1 cleavage assay, the DNA fragment covering the target site was amplified from thegenomic DNAs using PrimerSTAR HS DNA polymerase (TaKaRa, DR010A)with another primer pair set ssB2m-TAL-F/R, of which the sequences were: 5-GGAAGCTCATTTGGCCTGAAGGG-3 (forward) and 5-CTCTCAGAAGGTGCTACTAGACG-3 (reverse), respectively, and the product size was 565bp. After purification with a PCR cleanup kit (Axygen, AP-PCR-50), the purified PCR product was denatured and re-annealed in NEBuffer 2 (NEB)using a thermocycler. Thereafter, the re-annealed PCR products were digested with T7EN1 (NEB, M0302L) for 30 min at 37 °C and separated on a 2.5% agarose gel. The PCR products exhibiting additional band(s) after T7EN1 cleavage assay were sub-cloned into T vector (Takara, D103A). For each sample, the no less than 16 colonies were randomly picked up and sequenced using M13F(−47) primer.

### Immunohistochemical assay

The freshly collected tissues were fixed in 4% paraformaldehyde solution overnight, washed with PBS for 3~4 times and stored in 70% alcohol at 4 °C before use. The fixed tissues were embodied in paraffin and 5-μm sections were conventionally prepared. The sections were subjected to immunohistochemical staining using the monoclonal antibody of murine origin specific for porcine B2M protein (LifeSpan, Cat.: LS-1858) and HRP-conjugated rabbit anti-mouse IgG Fc antibody (PIERCE, USA) as the primary and secondary antibody, respectively. After stained with DAB kit (Zhongshan Biotech, China), the sections were examined and photographed under microscope (Olympus, Japan).

### Fluorescence-activated cell sorting (FACS) assay

Pig whole blood sample (~50 uL) was placed into Eppendorf tube containing 1 mL of PBS with 2 mM EDTA, and then subjected to centrifuge at 1200 rpm, room temperature for 5 min. After centrifuge, the blood cells were re-suspended with 5 mL of PBS. 1 mL of ACK solution was added into the sample and mixed thoroughly to lyse the red blood cells. The lysis process of red blood cells was repeated twice, and then the remaining blood cells were stained with 50 uL of FACS buffer (PBS with 2% (v/v) fetal bovine serum) containing 1:200 diluted FITC-conjugated SLA Class 1 antibody (GenTex, Cat.: GTX43353) or FITC-conjugated IgG1 isotype control antibody (eBioscience, Cat. No.: 8011-4714) at 4 °C for 30 min. Thereafter, 1 mL of FACS buffer was added and the sample subjected to centrifuge at 1400 rpm for 5 min. To wash the stained cells and eliminate the unbound antibodies, the blood cells were re-suspended with 1 mL of FACS buffer and spin at 1400 rpm for 5 min. This washing process was repeated twice, and then the stained blood cells were subjected to flow cytometry analysis.

### Skin grafting

Skin grafting was performed as previously described^31^,^32^. Briefly, Split-thickness skin grafts were made from the full thickness skin pieces surgically collected from pigs after anesthesia. The hair on the back of anesthetized recipient mice of FVBN strain was shaved off, and a 1 × 1 cm^2^ wound was made on the back by shaving off the dorsal skin carefully while leaving the subcutaneous vasculature intact. Porcine skin graft with the size of 1 × 1cm^2^ was planted into the wound and sutured with the host skin. To fasten the touch of skin graft with the wound, the porcine skin grafts were covered with a dressing of several layers of gauze pieces and secured with a transparent medical film dressing to provide a sustainable pressure. The survival of skin grafts was observed every other day, and the survival time was defined as the first day on which more than 90% area of skin grafts was necrotic as described by Wang *et al*.^32^.

## Additional Information

**How to cite this article**: Wang, Y. *et al*. Efficient generation of *B2m*-null pigs via injection of zygote with TALENs. *Sci. Rep.* **6**, 38854; doi: 10.1038/srep38854 (2016).

**Publisher’s note:** Springer Nature remains neutral with regard to jurisdictional claims in published maps and institutional affiliations.

## Supplementary Material

Supplementary Information

## Figures and Tables

**Table 1:** Summary of test embryo microinjection of *B2m*-TALEN.

Number of collected ovaries	Number of cultured COCs	Number of mature oocytes	Number of TALEN injection oocytes		Cleavage rate 48 h post activation		Blastocyst development rate 144 h post activation	
			Injected group	Untreated group	Injected group	Untreated group	Injected group	Untreated group
460	965	452	230	222	49.1% (113/230)	47.3% (105/222)	16.1% (37/230)	14.4% (32/222)

**Table 2:** Summary of alleles from test embryos with *B2m*-TALEN-mediated modifications.

Embryo No.	Target site mutation
#1	overlapped peaks
#2	WT
#3	overlapped peaks
#4	overlapped peaks
#5	del 10 bp; del 16 bp
#6	del 5 bp; del 7 bp

Del: deletion; WT: wild type.

**Table 3:** Summary of embryo microinjection with *B2m*-TALEN mRNAs.

	Injected materials	Injected embryos	Transferred embryos	Recipient amount	Established pregnancy	Piglets born
1st	*B2m*-TALEN mRNAs (10 ng/mL each)	40	40	2	0	0
2nd	*B2m*-TALEN mRNAs (10 ng/mL each)	26	26	1	0	0
3rd	*B2m*-TALEN mRNAs (10 ng/mL each)	59	57	2	2	5
4th	*B2m*-TALEN mRNAs (10 ng/mL each)	22	22	1	0	0
5th	*B2m*-TALEN mRNAs (10 ng/mL each)	41	41	2	1	2
Total		188	186	8	3	7

**Table 4:** Summary of allele from founders with *B2m*-TALEN-mediated modifications.

Founder No.	Target site mutations	Sequenced colonies
T1	del 1 bp (T)	1
	del 2 bp (TT)	11
	del 2 bp (AC)	1
	del 16 bp	3
T2	del 1 bp	5
	del 7 bp	4
	WT	6
T3	del 1 bp	9
	del 7 bp	3
	del 16 bp	2
	WT	5
T4	del 2 bp	7
	WT	14
T5	del 11 bp	4
	del 25 bp	13
T6	del 1 bp (A)	6
	WT	16
T7	WT	20

Del: deletion; WT: wild type.
