# Supplementary material for: Efficient generation of B2m-null pigs via injection of zygote with TALENs
Source: Sci Rep. 2016 Dec 16;6:38854. doi: 10.1038/srep38854 (PMC5159787; doi:10.1038/srep38854)
Supplement: Supplementary Information [file srep38854-s1.pdf]

Efficient generation of *B2m*-null pigs via injection of zygote with TALENs

Yong Wang[1, #], Yinan Du[2, 6, #], Xiaoyang Zhou[1], Lulu Wang[1], Jian Li[3], Fengchao Wang[4], Zhengen Huang[5], Xingxu Huang[2, 6, *], Hong Wei[1, *]

[1] Department of Laboratory Animal Science, College of Basic Medical Sciences, Third Military Medical University, Chongqing 400038, China.

[2] MOE Key Laboratory of Model Animal for Disease Study, Model Animal Research Center of Nanjing University, National Resource Center for Mutant Mice, Nanjing210061, China.

[3] Department of Immunology, College of Basic Medical Sciences, Third Military Medical University, Chongqing 400038, China.

[4] Institute of Combined Injury, College of Military Preventive Medicine, Third Military Medical University, Chongqing 400038, China.

[5] Research Institute of Burns, Southwest Hospital, Third Military Medical University, Chongqing 400038, China.

[6] School of Life Science and Technology, ShanghaiTech University, 100 Haike Rd., Pudong New Area, Shanghai 201210, China

[#] These authors contributed equally to this work.

*Correspondence: weihong63528@163.com (H.W), huangxx@shanghaitech.edu.cn (X.H.)

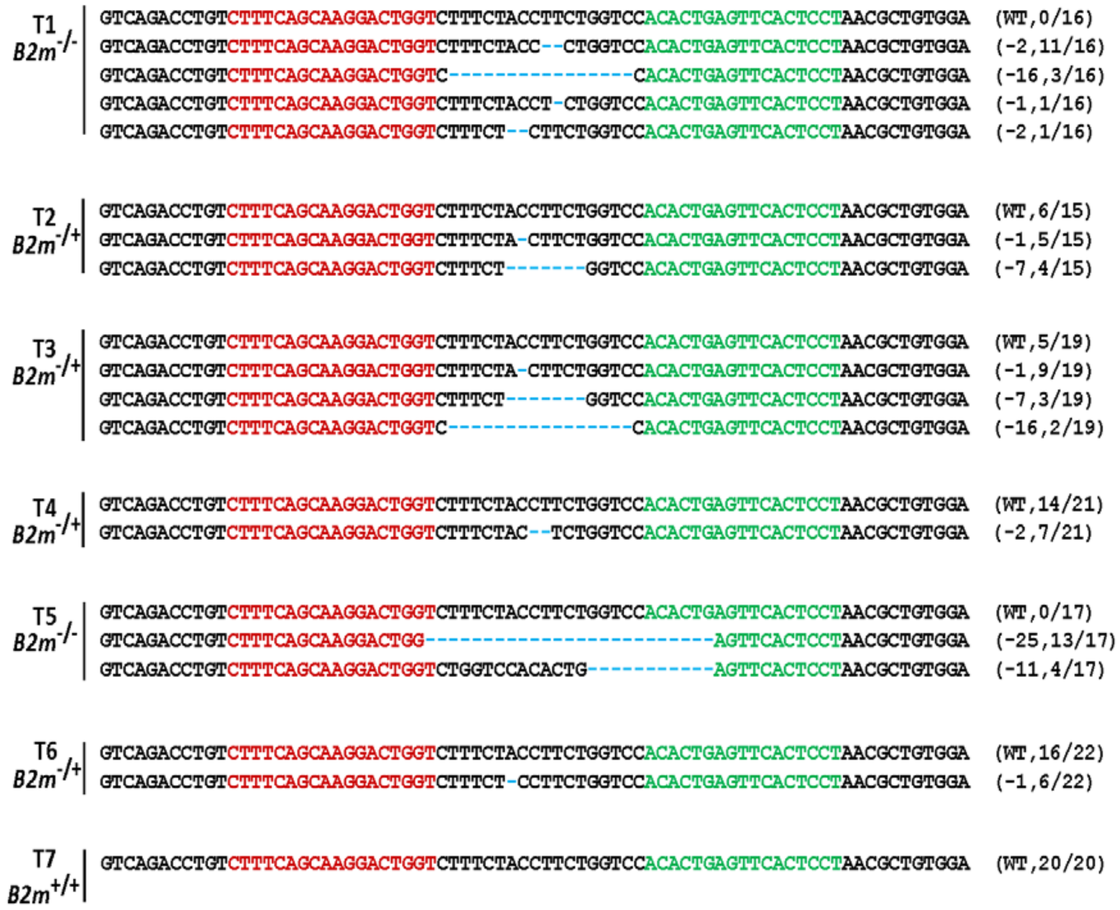

Figure S1 Sequencing results of modified *B2m* alleles in all the founder pigs.
